# Approaching OPC UA Publish–Subscribe in the Context of UDP-Based Multi-Channel Communication and Image Transmission

**DOI:** 10.3390/s21041296

**Published:** 2021-02-11

**Authors:** Alexandru Ioana, Camelia Burlacu, Adrian Korodi

**Affiliations:** Department of Automation and Applied Informatics, Faculty of Automation and Computers, University Politehnica Timisoara, 300223 Timisoara, Romania; tm.alexandru@yahoo.com (A.I.); camelia.burlacu@student.upt.ro (C.B.)

**Keywords:** interoperability, industrial internet of things, industrial protocols, OPC UA, publish–subscribe mechanism, image transmission, industry 4.0

## Abstract

The Open Platform Communication Unified Architecture (OPC UA) protocol is a key enabler of Industry 4.0 and Industrial Internet of Things (IIoT). OPC UA is already accepted by the industry and its presence is expected to reach more and more fields, applications, and hierarchical levels. Advances within the latest specifications are providing the opportunity to extend the capabilities and the applicability of the protocol, targeting better performances in terms of data volumes, speed, availability, footprint, and security. Continuing previous researches focusing on the publish–subscribe (pub/sub) mechanism and real-time constraints, the current study aims to consider higher data-volumes, approach the multi-channel User Datagram Protocol (UDP)-based communication, and analyze the robustness of the developed mechanism in the context of long-term data transmission. Consequently, the research proposes to extend the applicability of the OPC UA in the context of image transmission. Although highly needed, the image transmission after processing is currently beyond the reach of OPC UA or other legacy industrial protocols, being considered as a separate fraction in the industrial environment. The concept and developments are applied considering both the end-of-line industrial manufacturing process in the automotive sector and the car-to-infrastructure communication. Without special hardware constraints, the obtained results are proven to be appreciable, opening various future perspectives for image transmission using OPC UA.

## 1. Introduction

The manufacturing industry is continuously evolving towards a complete Industry 4.0 perspective. The evolution is taking steps in two directions—research and production line practical implementation. The research is following its natural trend with theoretical and practical approaches, focusing mostly on a high pace innovative actions in the Industrial Internet of Things (IIoT). The practical industrial development is always slowed down by legacy equipment, solutions, and procedures, but at the same time, it is always pushed forward by necessities-based, quickly developed new entities and services that, at least for the short-term, are fulfilling the needs for evolution. These new entities and services are not always the best or complete options and often are not seeing the big picture in terms of research status. However, they are clear improvements regarding the current status in the manufacturing industry. These examples are widespread throughout the industry and chronology and the recent years’ high pace developments are showing more and more situations. The need for data integration and lack of a complete IIoT picture often implements a local legacy protocol that is integrated using centralizing Open Platform Communication (OPC) servers without considering direct Open Platform Communication Unified Architecture (OPC UA) interfacing or local OPC UA wrappers [1,2]. The need for collaborative robots (cobots) alignment to functional requirements often leads to fast solutions within the companies that are completely lacking industrial protocol, robustness, compatibility, and security (e.g., string-based basic particular communication strategy developed within small research and development teams inside companies). This is not considering the possibility of OPC UA within cobots [3]. The data gathering strategies on the operational technology level are usually implemented as classic historian solutions with various overestimated but small capacity solutions, without considering a proactive historian possibility (e.g., [4]). The information technology level process data integration is mostly regarded as repetitive rudimentary file export–import algorithm, or at best representational state transfer (REST) based or proprietary connector-based solutions, while an evolved industrial protocol would offer clear benefits. For best solutions, the research has to move more clearly and faster to higher technology readiness levels (TRLs), respectively its connection to the industry has to be enforced.

The conceptual approach and applicability of the multi-channel UDP communication strategy alongside the results and observations of the multiple experiments with the publish–subscribe (pub/sub) mechanism in terms of capacity and stability may prove useful in future integrations of the OPC UA protocol in different architectures.

Papers such as [5] are analyzing the compliance of industry standards through OPC UA. The latest OPC UA specifications (e.g., [6]) are allowing to step to the next level with OPC UA and to approach the publish–subscribe mechanism. This way the capabilities of the protocol can be extended, and better performances are reachable regarding volumes, speed, availability, footprint, and security. The OPC UA publish–subscribe mechanism is developed successfully in [7,8], allowing the expansion of the protocol applicability. However, the research has to evolve and provide a solution for OPC UA publish–subscribe based image transmission that would allow complete integration of the protocol in the industry. Moreover, the OPC UA publish–subscribe solution from previous researches has to prove robustness in the context of long-term data transmission.

In the above-mentioned context, the objectives of the current research are as follows:

- to continue research [7] focusing on publish–subscribe mechanism and real-time constraints, and to consider and analyze higher data-volumes that would mean long-term single data transmission;
- to approach the multi-channel User Datagram Protocol (UDP)-based communication and to analyze the developed mechanism, purposing to reduce the duration of the data transfer;
- to extend the applicability of the OPC UA in the context of image transmission;
- to apply the developed mechanism without using any special hardware constraints and analyze the results in case studies referring to the automotive industrial manufacturing end-of-line testing and the car-to-infrastructure communication.

The study presents in the second section relevant insights from the research world and from the industry. The third section presents the aspects referring to the OPC UA publish–subscribe mechanism in the industrial research context and the image processing in the OPC UA context followed by the architecture and the implementation of the OPC UA multi-channel UDP based communication. The fourth section is advancing with the presentation of the developed case studies and analysis of the obtained results. The final section discusses and concludes the research.

## 2. Related Research

Image processing in the industry emerged from necessity and it was adopted without considering a complete link of the results towards industrial protocol and the production process. The general current status of communication with other entities is a manufacturing execution system (MES) binary request-approval based procedure execution and bit-wise result storage, respectively the images storage and transportation is realized without an industrial protocol. However, the image processing itself is strongly related to production lines. In [9], the research presented a diagnosis of the hydraulic axial pump system using a conversion of signals to images that is conducted via continuous wavelet transform, respectively, the extraction of the feature from the transformed time–frequency images. In [10], the authors approached an image-processing solution and deep learning to detect deformation on pantograph contact strip of railway vehicles. In [11], the authors presented a low-cost image-processing solution based on Open-Source Computer Vision Library (OpenCV) that is used to detect defects in automotive parts manufacturing, particularly defects as faulty, missing, or extra pins, faulty clips, board cracks, and electronic control units (ECUs). Most studies are considering that the industrial process and the image transmission as separate fractions. In the augmented reality domain, the study in [12] proposed Node-RED and Message Queuing Telemetry Transport (MQTT) for communicating with the mechatronic devices, but the image-related issues are concentrated on the mobile device within the iOS application.

In addition to the production process itself, the automotive industry focuses on new concepts as autonomous and improved driving. These concepts are strongly linked to image processing, car-to-infrastructure and car-to-car communication, and safety procedures. Great progress has been made in image processing techniques as an essential aspect of autonomous driving [13]. Moreover, image processing is researched effectively to improve driving and safety and detect damages (cracks) in the infrastructure, as depicted in [14]. Accident detection based on images from the infrastructure was considered in [15]. The intelligent roadside devices are taking over and processing traffic images using YOLO–CA (a YOLO – You Only Look Once - deep-learning model for car accidents), and results are foreseen to be sent to a central system for rescuing purposes, and elementary signals to cars within the traffic. The study in [15] focused on image processing but addressed the area of car-to-infrastructure communication. In [16], the authors presented a queue length estimation based on image processing in the context of car-to-infrastructure communication. The results were presented using only simulations. Obviously, car-to-infrastructure communication, in the context of horizontal and vertical interoperability, requires an industrial protocol, and papers such as [7] and [17] detailed the car-to-infrastructure communication approach based on OPC UA.

## 3. Materials and Methods

The current section focuses on the applicability of the OPC UA protocol alongside the publish–subscribe mechanism in the industry, on different types of requirements, and on possible advantages gained from the integration with other technologies in various use-cases. The authors propose an application for image transmission using the publish–subscribe paradigm in multi-channel, real-time communication.

### 3.1. OPC UA Publish–Subscribe in the Industrial Research Context

OPC UA is a highly adopted protocol in the industry, being used worldwide and providing high reliability and interoperability for various systems, allowing the integration of multiple technologies and features. With [6], the publish–subscribe paradigm was introduced, expanding the capabilities of the standard and widening the areas of applicability toward different domains. A more complex set of requirements was able to be accomplished, scenarios such as controller-to-controller communication and other time-constrained operation have been achieved. The literature presents few consistent research studies regarding the OPC UA publish–subscribe concept. The authors in [7] presented research that analyzed the real-time requirements from different perspectives in the context of the OPC UA publish–subscribe mechanism, and the concept that is targeting different domains has been implemented. Other studies have targeted the exploration of the OPC UA publish–subscribe mechanism in comparison with similar paradigms from different communication protocols used in other domains, and architectural insights and improvements have been suggested for specific use-cases from the industry [8].

In terms of hardware devices used in research and in the industry for implementing and testing applications based on the OPC UA publish–subscribe mechanism, a range of different types of devices can be observed. However, with the real-time capabilities of the mechanism, the tendency is toward embedded devices with low resources and low cost, based on the controller-to controller scenario specific to pub/sub. In [18], multiple Xilinx boards were used for running a publisher and a subscriber that exchange UA Datagram Protocol (UADP) network messages, and time analysis of the operations was performed. In [19], for the exchange of information, the OPC UA entities were implemented on Raspberry PI Model 3B+, from different open-source stacks, and with efficiency measurements being performed. In [8], the authors implemented and tested multiple OPC UA publishers and subscribers on devices with high resources and also on embedded devices with the objective of performing synchronization regarding the information exchange among devices.

Based on [7], the time-sensitive networking (TSN) technology represents a solution for time-critical information exchange over Ethernet, taking into consideration real-time constraints and being of use for both sensors-to-cloud communication or controller-to-controller communication. The TSN is specific to the data link layer, and through a set of particular standards, it offers time guarantees for certain operations across the network. As stated in [20] and [21], OPC UA and TSN are foreseen also to the field level, respectively. The study in [22] emphasized the real-time issue and the applicability of the concept on the field devices, but it provides only simulations. In the case of OPC UA publish–subscribe mechanism, the integration of TSN technology represents the next step in achieving real-time behavior, increased efficiency, and quality of service. Studies have been performed towards the interaction of OPC UA and TSN, and the results are proving that time synchronization, low latency times, and flexibility can be achieved by adopting different standards to the well-known mechanism that is present in the industry [18,23].

With [6] still being recent, there are directions that can be explored toward the efficiency and reliability of the concept, and new applications with particular features can be developed for solving industry-specific challenges. With the increasing number of network devices present in more Industrial Internet of Things specific applications, the multitude of operations that need to be performed is increasing, and as stated in [24], the management and extraction of data from multiple and distributed sources is becoming decisive. All the implied entities that are present in such complex architectures need to evolve in such a way that all demands have to meet the expectations in terms of reliability, robustness, and efficiency. Mechanisms such as OPC UA publish–subscribe are increasing the capabilities of convoluted systems, however, in industrial applications or in research, there is a projection towards a more varied set of functionalities and requirements for any IIoT application in the near future. Moreover, all the implied technologies must be analyzed in different scenarios. It makes sense to think about new purposes and use-cases for solving or improving the existing solutions for certain services. In this context, the authors chose a scenario that is feasible for analyzing the capabilities of the publish–subscribe mechanism from different perspectives, and implemented an image transmission application based on the OPC UA protocol.

### 3.2. Image-Transmission over OPC UA Publish–Subscribe Concept

Image transmission and image processing are concepts that are more and more present in the IIoT applications. With growing capabilities over the Ethernet, it is expected to become part of processes existing in smart factories, the manufacturing industry, and other domains such as automotive or aeronautics. With OPC UA as a key link in interoperability and having already integrated multiple technologies at different levels of the Open Systems Interconnection (OSI) model, new features as image transmission could prove useful in certain scenarios.

Image processing has an important role in developing safety applications in the automotive industry and for intelligent traffic systems. With the autonomous driving concept being of high interest nowadays, the majority of automotive manufacturers are implementing modules based on image processing. The capabilities of inter-car communication and intra-car communication, alongside vehicle-to-infrastructure communication, are evolving at a high pace. In both cases of smart-infrastructure and smart-cars, pedestrian detection and fast information-exchange over the Ethernet are vital concepts. Studies have been performed toward the integration between OPC UA as a protocol with potential for smart-infrastructures and other communication protocols specific to the automotive domain and solutions that could route information through publish–subscribe mechanisms, including the OPC UA pub/sub have been developed for these scenarios [7,17]. The potential towards the extension of the usage for OPC UA is expected to determine future applicability in the safety modules that are based on image processing and image transmission.

In the automotive manufacturing industry, image processing is frequently used. As exemplified in [11], during the manufacturing of ECU boards, automatic optical inspection (AOI) is performed at the end-of-line testing and packaging. Referring strictly to the end-of-line testing and packaging, tens of thousands of products are tested daily in a company using image processing for various defects, and the packaging process is also assured using image processing.

**Figure 1:**
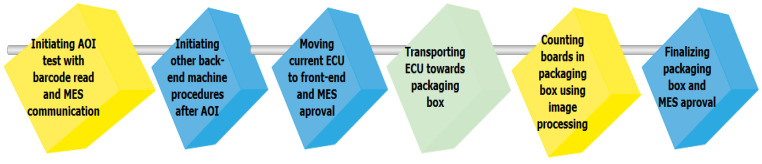
End-of-line electronic control unit (ECU) testing using image processing in automotive manufacturing.

The results of the image processing solutions include also images. Because the bitwise/tag-based results are integrated with the MES communication usually without any industrial protocol, the concluded and locally reported images are transferred from time to time in a rudimentary manner from one place to another. An end-of-line process flow is presented in Figure 1, where image processing is used at the end-of-line testing and packaging in automotive parts manufacturing. After pins are inserted, and the ECU enclosure is positioned, the ECUs are tested using AOI for defects at pins (e.g., bent, misplaced, missing, or extra pins), at clips (improper placement or broken clips), or board cracks. The AOI solution is communicating with the MES requesting approval to begin the testing and transmitting a bitwise result if the board is faulty or passed. After the AOI is performed, the best solution regarding Industry 4.0 concepts would be to integrate the OPC UA protocol for the image processing solution. However, OPC UA would have to assure also the real-time, speed, and volume requirements. Based on researches of [7] and [8], the bitwise/tag-based communication would assure the real-time constraints and the publish–subscribe mechanism. The purpose is to elevate the OPC UA publish–subscribe mechanism from the previous researches to fit longer-term, higher volumes, and faster data movement, which are necessary requirements for image transmissions in the context of complete integration with the process flow. This way, complete vertical and horizontal interoperability could be achieved using OPC UA. As depicted in Figure 1, the boards are transported afterward and inserted into the packaging boxes. The ECU counting inside the packaging boxes is realized also using image processing. After reaching a box capacity, before closing and sending it to the client, a final image with all the boards inside is stored. The same issue regarding complete OPC UA interoperability would be needed also in this situation, where the images are usually larger but the times between image transmissions are also increased.

One of the use-cases needed to be tested in terms of performance against the image transmission application is the efficiency of processing an image and the extraction of high-interest objects by a neuronal network and publishing the objects to a subscriber in real-time. This type of use-case can provide some time delivery limits regarding some of the minimum and necessary information that could be obtained from an image. This type of use-case is detailed in Section 4.

For the processing operations, the YOLO v3 model [25] was used. YOLO involves a single deep convolutional neural network (called DarkNet) that splits the input image into a grid of cells and each cell directly predicts a bounding box and object classification. The result is a large number of candidates bounding boxes that are consolidated into a final prediction by a post-processing step. For significantly reducing any false detection, YOLO was trained on the COCO dataset and this model could detect objects for 80 classes.

For different operations specific to the targeted image for transmission, the Open Source Computer Vision Library (OpenCV) was used, popular in the industry as a solution specialized in real-time computer vision. OpenCV is cross-platform and free to use under the open-source Apache2 License and supports Torch/PyTorch, TensorFlow, and Caffe deep learning software. The application areas for OpenCV are facial recognition, human–computer interaction, 2D and 3D feature toolkits, motion tracking, mobile robotics, and image segmentation.

**Figure 2:**
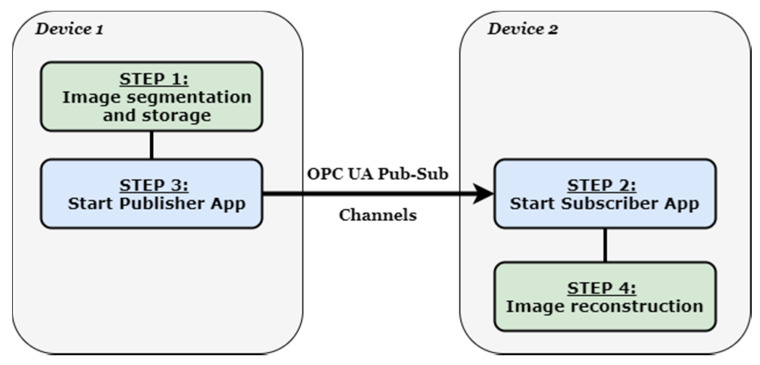
Open Platform Communication Unified Architecture (OPC UA) publish–subscribe image-transmission steps.

The image transmission application is formed by two different instances specific to the OPC UA publisher and to the OPC UA subscriber, and it is focused on sending an image from a device through the OPC UA pub/sub mechanism. There are three versions of the application, each one is targeting a different case-study in Section 4 and have achieved different performances. The main objective is to send an image by publishing each pixel and rebuilding the image at the receiver side after the subscriber has obtained and store all pixels from the publisher. The speed of the full transmission and the quality of the received image are the main parameters that prove the reliability, efficiency, and feasibility of the concept. The application is tested in different scenarios and different images are sent, each one with particularities in terms of resolution. For all three versions of the application, the publishing interval is set to different values for obtaining different time intervals for delivery and for analyzing how the speed of the transmission could influence the quality of the image. The publisher entity and the subscriber entity are configured to exchange DataSetMessages [6], each one containing the value of one pixel in byte image format, and the value is stored as an 8-bit integer with a range between 0 and 255. The DataSetMessages are created by the DataSetWriter component of the publisher. The transport protocol used for the pub/sub transmission is User Data Protocol (UDP). The concept is based on four main steps for complete image transmission and can be observed in Figure 2.

The time duration of the transmissions and other operations specific to each scenario have been measured using specially developed test functions that use Linux timers. In terms of networking, the concept has been tested in networks with different capabilities (2.4 GHz and 5 GHz). With the OPC UA pub/sub mechanism being real-time oriented but not capable of offering time guarantees without the integration of TSN technology, in the case of fast transmissions over long time periods, desynchronization may occur between the publishing and receiving operations that are running on the two devices. The pub/sub mechanism design focuses primarily on one-to-many scenarios, however with the capacity of achieving real-time demands and in cases where desynchronization and latency are desired to be avoided, multiple completely individual pub/sub channels can provide a scalable solution for a range of multiple industrial operations that previously could not be solved in a feasible way by the classic server–client paradigm. In the case of multi-channel transmission, efficient communication between entities is important and studies have been performed toward algorithms that can improve the information exchange [26]. However, in the current implementation, independent channels have been developed for minimizing the transmission time with all channels transmitting at a high recurrence, obtaining a predictable behavior based on the targeted quantity of information desired to be delivered (the pixel values of a certain image). Security in the context of IIoT represents an important topic in the research community [27,28] and from the security point of view, OPC UA provides mechanisms at different layers on the OSI model that assured over time the growth of interoperability for the protocol, so the multi-channel approach may not present increased security risks even toward a case such as image transmission.

For the implementation of the OPC UA entities, the authors have taken into consideration multiple OPC UA open-source stacks implemented in different programming languages for the current study. Based on the implementation reliability that was present in various research projects [7,8,29], and on studies regarding the capabilities and the efficiency of the OPC UA stacks [19], the open62541 SDK [30] was selected for the implementation of the OPC UA publisher and the OPC UA subscriber on both devices.

### 3.3. Architecture and Implementation

The OPC UA image transmission application is composed of two parts—one as an OPC UA publisher that is specific to the device that wants to transmit the image, and one as an OPC UA subscriber that runs on the device targeted to receive the image. In addition to the two different parts, the additional steps required before and after the transmission of the image, such as the segmentation of the targeted image and the reconstruction on the receiver device, are also important and are establishing different operations that are required to be executed following a specific order.

**Figure 3:**
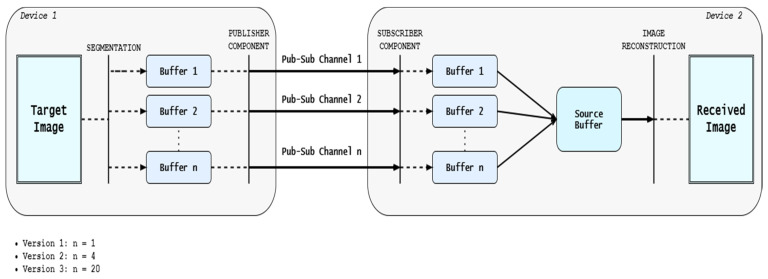
General system architecture.

As stated in the previous section, the main four steps of the application are image segmentation and storage, starting the subscriber app, starting the publisher app, and image reconstruction. There are different versions of the application, associated with different case studies and under specific configurations. The first step regarding the image segmentation and storage is splitting the image into approximately equal parts, each part with the same number of pixels. Afterward, it creates a different buffer for each part that is accessible by the OPC UA publisher at step 3 of the process. The splitting of the image is done based on the number of pub/sub channels that are desired to be used for the transmission, respectively based on the size of the image to be transmitted. Step 4, the image reconstruction on the subscriber side, is a reverse process of step 1. The subscriber receives various parts of the image on different pub/sub channels at the same time and stores them in its own buffers. After the transmission is carried out, the buffers are used to construct an image specific pixel file where each received part of the image is placed in the correct order. If the transmission has been conducted correctly for all the involved channels, and if the pixel values have been placed in the correct order, the image can be reconstructed on the receiving device with the same characteristics as the original one. The architecture of the system is presented in Figure 3, with all the operations happening during the execution of the four main steps of the OPC UA image-transmission application.

The publishing process and the receiving process are synchronized to transmit and receive pixel values at certain time intervals. As stated in [8], while the transmission is done over UDP, without TSN technology, the devices are not connected to a common time base. There are two devices sharing information, but each is managed based on its own time frame and with different definitions of time intervals. This scenario might affect the desired behavior of the application. When the publishing time interval is expected to publish values in under 10 milliseconds, without the possibility of losing some of the values, as each value represents a pixel and each pixel sent only once, and without a mechanism for preventing message losses, the impact toward the final result is influenced by the robustness of the used methods and by the capabilities of the network. However, even if a couple of pixels might not alter decisively the quality of the received image, having in mind that multiple parts of the image are transmitted simultaneously through different pub/sub channels and that afterward the content of the buffers are forming a single file for the pixels, the development of a safety mechanism for this type of cases was necessary. The mechanism is specific only to the Subscriber part of the application, and based on the resolution of the image desired to be received, each buffer specific to every pub/sub channel is tested and every receiving operation is counted. At the end of the transmission, if the number of values that are present in each buffer does not match the expected number of pixel values, the mechanism adds default values until the buffer is full. The advantages of this approach are that the image can be reconstructed even if it was not entirely transmitted. Moreover, based on the quality of the reconstructed image, the observer can identify on which pub/sub channels have the transmission suffered desynchronizations or losses, and when they have occurred, considering the approximate time-frame expected for the transmission.

The publisher and subscriber parts of the application are implemented based on [6], and for each pub/sub channel, the specific components are present on both sides. As stated in [7], the publisher side is responsible for creating the DataSetMessages that are sent to the subscriber at a time interval that is configured in the WriterGroup component. The payload of the DataSetMessage is the value of a pixel taken from the buffers that are storing the values after the segmentation of the image is finished. After the sending of one value to the subscriber, a new DataSetMessage is created and sent with the next value from the buffer. This way, it is crucial for the subscriber to receive all the messages in order to obtain an identical reconstructed image quality with the original image. The subscriber receives the messages periodically and extracts the payload from the only DataSetMessage field present, keeping the structure of the messages as simple as possible. Even in the case of multiple channels with multiple publishers and subscribers involved in the transmission, the targeted design is one-to-one communication, so the subscriber entity can be implemented similarly to the way described in [8], skipping some of the filtering steps and obtaining a faster execution time, needed in the cases of high-speed transmissions.

The different versions of the OPC UA image transmission application implement a different number of pub/sub channels and storage buffers for both devices and target the following for observation and analysis:

- the robustness of the OPC UA publish–subscribe mechanism;
- the speed of the transmission for images with different resolutions;
- the quality of the received and reconstructed images in comparison to the original.

Other major objectives include the following:

- the examination of the behavior in the context of different networks with different capabilities;
- the achievement of a stable solution that can deliver identical images in time periods that could prove useful in the industry;
- the engagement of the concept in authentic and valid industry-based scenarios with a clear idea regarding the advantages and disadvantages of the OPC UA publish–subscribe mechanism.

## 4. Case Study and Results 

The OPC UA pub/sub mechanism can produce complex applications with real-time requirements and can expand the current use of the protocol towards other functionalities or other domains. The authors are not aware of other work toward image transmission through OPC UA. Based on the popularity of the protocol in the industry and with the evolution of the integrated technologies and mechanisms, these implementations with certain achieved goals can resolve or improve current scenarios and provide a step forward toward interconnectivity in the IIoT context.

### 4.1. Initial Case-Study for Performance Evaluation in the Context of Image Processing

For a primary analysis regarding the usage of the OPC UA publish–subscribe mechanism in the context of image processing, the chosen use case was the object detection and transmission from a publisher of messages, based on the presence of objects of interest, to a subscriber located on a different device. The goal of the case study was to integrate the mechanism to a popular scenario from the industry and determine a time interval on which the operation can be completed. YOLO was used for the detection of eight objects of interest within an image and eight messages were sent signaling the presence or absence of the objects to a second device.

The completion of the full operation was done in 2.11 s; the majority of the time was consumed for the detection of the features within the image. The publishing interval for the messages between the publisher entity and subscriber entity being 1 millisecond for a message (8 milliseconds for all the messages). The execution time for the whole operation is representing an indicative target for the following case studies, where the subscriber will receive the whole image (not only the message depicting the presence of the objects within the image), so the transmitted information is under a more complex form.

### 4.2. Case Study 1—Image Transmission over One Pub/Sub Channel

**Figure 4:**
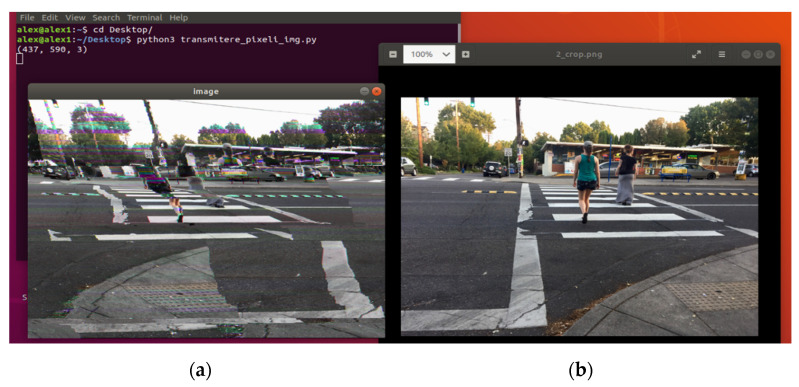
Comparison between the received image and (**a**) the target image and (**b**) at 1 ms/pixel value recurrence (phase 1).

**Figure 5:**
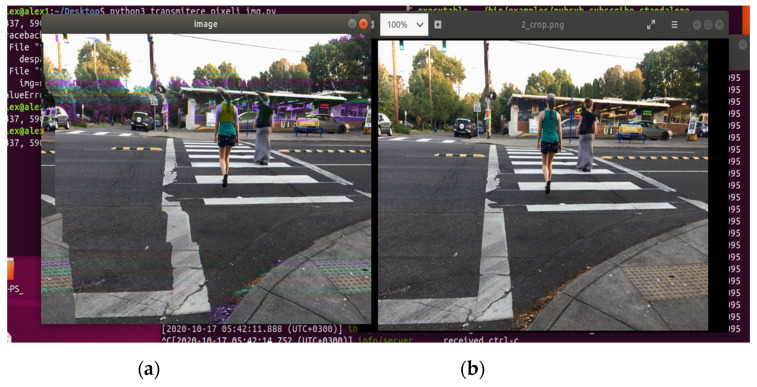
Comparison between the received image and (**a**) the target image and (**b**) at 5 ms/pixel value recurrence (phase 1).

The full transmission of an image over OPC UA publish–subscribe was targeted to determine the feasibility of the concept and to establish the relation between the speed of transmission and the quality of the received image. In an initial worst-case type of scenario shaping the first phase of the case study, using only one pub/sub channel, a medium-large size color picture was transmitted. The scenario used a classic Ethernet connection (without TSN technology), in order to determine any possible impediments and desynchronization problems that can occur and identifying the causes and solutions for such obstacles. In the case of Figure 4 and Figure 5, a Wi-Fi connection was used, to obtain a worst-case physical support scenario.

The publishing intervals of the transmissions were established for stability reasons between 1 millisecond (1 ms/pixel value) and 5 milliseconds, with the main goal of achieving an image under being as close as possible to the original target. In Figure 4 and Figure 5, the quality of the receiving images can be observed in comparison to the original image.

With 773,490-pixel values (for the target picture from Figure 4 and Figure 5) sent between the publisher and the subscriber at different recurrences, the results prove that the implementation is capable of lengthy transmission (approximately 12.9 min at 1 ms/pixel value and 64.5 min at 5 ms/pixel value) to deliver an image to the second device, proving the robustness of the pub/sub mechanism over UDP. The results can be categorized as better and better with an increase of the publishing time interval. The explanation is that, with the values being sent at a lower speed, any desynchronization between the two implied devices, determined by time management or network instability, will produce less damage to the quality of the received image. During the length of the desynchronization, with fewer values being sent in a fixed time window, fewer values will be lost, therefore, at 5 ms/value, the receiving picture will become visible closer in terms of quality to the original target. However, even if the quality of the received image is improving, the time of the transmission is far from any bearable limit present in the industry, so future tests toward the initial phase of the case studies are not useful at this point.

**Figure 6:**
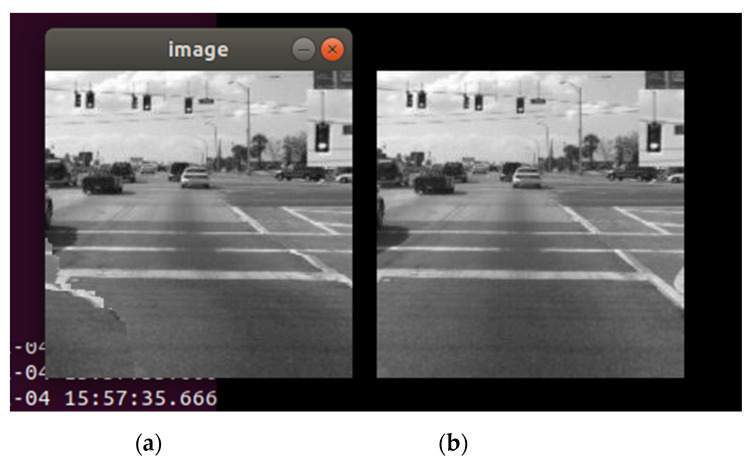
Comparison between the received image and (**a**) the target image and (**b**) at 1 ms/pixel value recurrence (phase 2).

**Figure 7:**
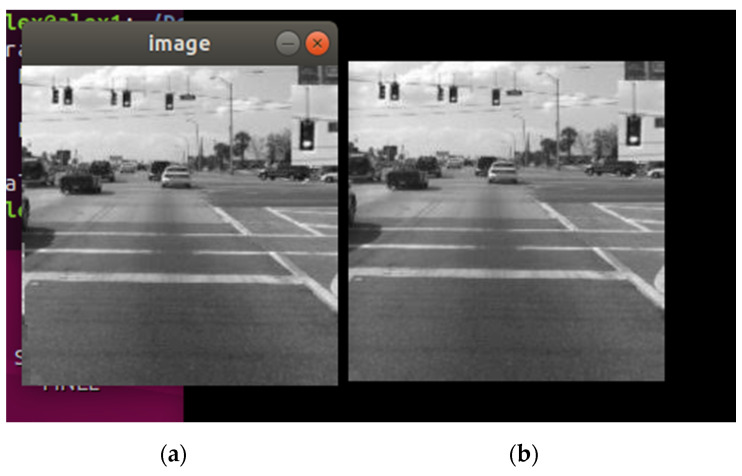
Comparison between the received image and (**a**) the target image and (**b**) at 4 ms/pixel value recurrence (phase 2).

The second phase of the case study is focusing on a more probable industry scenario, where the target image is at a lower resolution and size and under black-and-white format, for a decrease of the number of pixel values needed to be sent, with the goal of achieving a more relevant time interval for the full transmission to the second device. The selected image contains 46,225-pixel values as payload and the implementation of the pub/sub mechanism is identical to the previous phase. The results can be observed in Figure 6 and Figure 7.

For the second phase of the case study, an identical image was received using a publishing interval of 4 milliseconds. With fewer amounts of values to be sent, a faster transmission time made the probability of desynchronization smaller even at the same recurrences as phase 1. It can be observed that in Figure 6, the desynchronization appeared in the third quarter of the image, and the alteration of the received image is lesser in comparison to the received image from phase 1 (Figure 4). The same effect made it possible to obtain an identical image at a higher speed than in the initial phase, using the exact same implementation. Given the target image used at phase 2, the time of the transmission was improved significantly—approximately 47 s at a publishing interval of 1 millisecond (1 ms/pixel value) and approximately 3.12 min at a publishing interval of 4 milliseconds. Phase 2 of the case study concluded that the OPC UA pub/sub mechanism is offering the possibility for image transmission in fixed time periods and even with stability problems over lengthy transmissions, the communication between the publisher and subscriber is not disrupted even at high publishing rates. In the context of industrial images (with less size, colors, and resolution) it can represent a solution with performances that can be improved.

### 4.3. Case Study 2—Image Transmission over Four Pub/Sub Channels

With the assurance that the OPC UA pub/sub mechanism can deliver an image from one device to the other without any alteration, the improvements that can be done are towards the delivery time of the full transmission. Among the factors that can improve the time of the transmission, in addition to the target image itself, is the multi-channel transmission, which will deliver different segments of the image in parallel, decreasing the number of pixel values needed to be sent per channel and therefore decreasing the probability of desynchronization and increasing the probability of receiving an identical image on the subscriber side at even higher recurrence. For the process to be successful, four different pub/sub channels were implemented working independently. The four publisher entities exchange information with the four subscribers implemented on the second device on two multicast addresses, each publisher using a different port. The publishing intervals are configured to be the same, for the length of the four transmissions to be approximately the same.

The results of the experiment confirmed the initial conclusions from case study 1. The received image from the second device was identical to the target image (the same as in Figure 7) at a publishing interval of 1 millisecond (1 millisecond/pixel value for all the four channels), obtaining a full transmission in approximately 12 s.

In addition to confirming the already observed behaviors, the current case study provides a stable solution capable of transmitting an image from one device to the other over the OPC UA pub/sub mechanism, proving that the multi-channel transmission concept can improve the performances of the application, increasing the stability even at high rates transmissions, and offering a scalable method of implementation with potential to achieve the time constraints needed in the industry for image transmission.

### 4.4. Case Study 3—Image Transmission over 20 Pub/Sub Channels

The current case study aims to obtain a feasible transmission time, demonstrating the scalability of the multi-channel transmission over OPC UA publish–subscribe mechanism, integrate the concept in scenarios that are present in the industry using relevant images within industrial processes, and test the impact of the network capacity in the context of the ongoing implementation.

Case study 3 refers to three scenarios in the context of OPC UA publish–subscribe image transmission, which are as follows:

- the first scenario consists of the previously discussed car-to-infrastructure communication;
- the second scenario analyses the transmission of the full package box status image in the automotive manufacturing end-of-line testing;
- the third scenario focuses on the automotive manufacturing production line, on the ECU automatic optical inspection negative fault detection test results transmission.

The main goal, in terms of the delivery time of the image from one device to the others, was to succeed in the operation in:

- under 3 s for the car-to-infrastructure communication;
- under 12 s for the end-of-line packaging boxes in automotive manufacturing;
- under 3 s for an image representing a negative result in the ECU fault detection.

The previous case study scenarios, where car-to-infrastructure communication context was considered, is extended first for the case study 3 implementation. The results of the current version of the application, which uses 20 channels, provided a delivery time of approximately 2.4 s for the same images used in case studies 1 and 2 (Figure 6 and Figure 7), with a publishing interval of 1 millisecond (1 millisecond/pixel value for all the 20 channels). The obtained image was identical to the target one (same result as seen in Figure 7). The scalability of the multi-channel transmission concept is confirmed, allowing multiple configurations for different scenarios in future developments of the application.

The concept is tested further using industrial manufacturing scenarios, where image processing is already part of the production process, and its applicability is continuously extending. The previously described automotive parts manufacturing End-Of-Line (EOL) ECU automatic optical inspection process, part of the final product testing process, will be divided into two scenarios. The two scenarios are representing two steps within the EOL testing where AOI is used.

**Figure 8:**
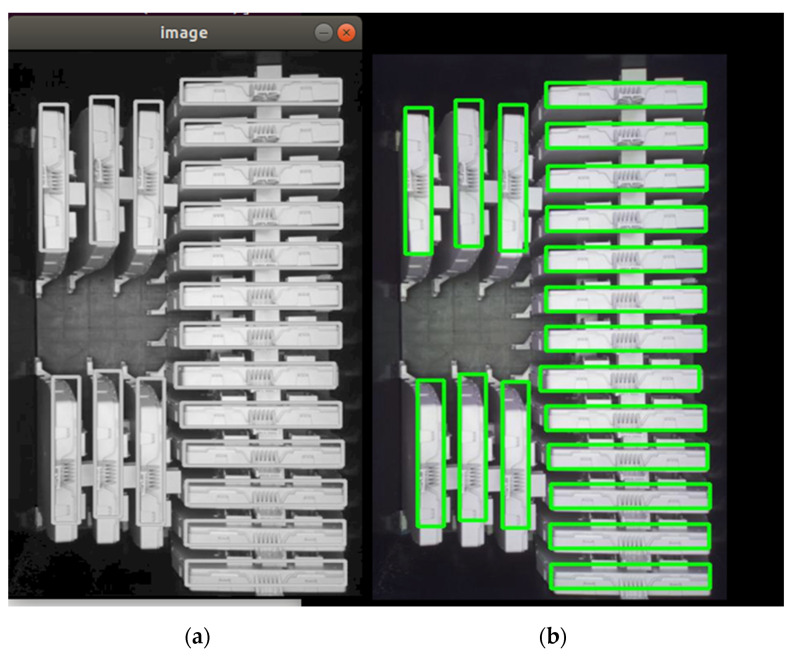
OPC UA publish–subscribe, 20-channel image transmission for packaging boxes in the automotive manufacturing: (**a**) the received image and (**b**) the target image.

First, case study 3 is focused on the final packaging boxes from the end of the production and testing process, consisting of ECUs that will be delivered to the client. After the continuous counting of the ECUs that are placed in the boxes, when the counting limit for the chosen box is reached, the MES is notified that the box packaging is finished and the final image is stored locally (see Figure 8b). Considering the box filling production cycle, the specific execution times in the manufacturing line are the following: a new ECU is inserted the box after a minimum of 12 s; the detection of a new box by the optical inspection solution is around 6 s; and a complete filling, depending on the box and the ECUs, is more than 2.5 min.

After implementing the OPC UA publish–subscribe, multi-channel image transmission, the results are encouraging. The target image presented in Figure 8b is converted in grayscale and transmitted without any faults to the destination. The received image is presented in Figure 8a.

For the full transmission of the image represented in Figure 8, a time of 7.85 s has been obtained. Therefore, the solution would allow from the time perspective even the transmission of each image for each inserted board in the box.

**Figure 9:**
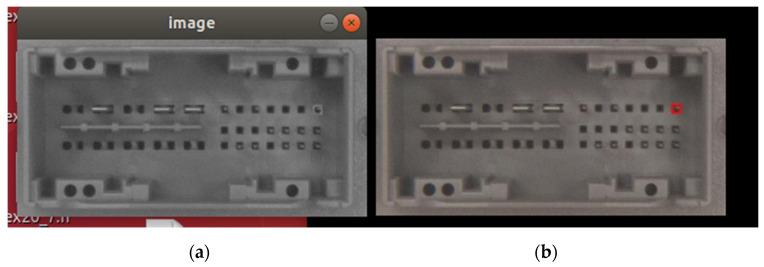
OPC UA publish–subscribe, 20-channel image transmission for ECU automatic optical inspection process in the automotive manufacturing: (**a**) the received image and (**b**) the target image.

The final case study 3 scenario is oriented toward a previous step within the EOL testing of ECUs in the automotive manufacturing, the automatic optical boards inspection, detecting ECU defects at pins, clips, or surface cracks [11]. The results of [11] present the image processing-based detection of defects realized within 6.5 s. The passed/failed bit result is transmitted to the MES under the required time limit. Considering the duration of all other procedures that are taking place in the production line, moving from one board to the next in the automatic optical inspection process would grant around 12 s to transmit the resulting images inside each board testing cycle. Only defects (negative fault detection results) are stored and would require to be transmitted, respectively, the negative test results are usually not more than 1–2 images because a stored image may contain more than one defects (e.g., the image from Figure 9 illustrates a connector that may contain more than 30 defects at pins).

After implementing the 20-channel-based OPC UA publish–subscribe image transmission, the results are again encouraging. The target image presented in Figure 9b is converted in grayscale and transmitted without any faults to the destination. The received image is presented in Figure 9a.

For the full transmission of the image represented in Figure 9, a time of 2.52 s has been obtained. Therefore, considering the 12 s available time within each board testing cycle, a number of around four images can be transmitted using the 20-channel solution.

The concept was tested in different networks of 2.4 GHz and 5 GHz. The probability of desynchronization can appear on both networks as long as the two devices are communicating over UDP and do not synchronize at the data link layer through dedicated standards as in the case of TSN technology. However, the probability itself cannot be properly calculated with so many factors that are in place, but efficiency of 100% was achieved on the 5GHz network with the current version of the application. It is expected that in industrial scenarios, the networking problems regarding the stability to be monitored and managed with additional instruments, assuring a high quality for all the involved services. Future studies may show a more complete view of the impact between OPC UA pub/sub applications and industrial networks with different types of devices as the OPC UA pub/sub mechanism shall gain acceptance in the automation field.

### 4.5. Results

The implementation of the OPC UA publish–subscribe image transmission application was successful. Each case study generated important conclusions regarding the stability and robustness of the publish–subscribe mechanism, the factors that can influence the transmission in an industrial context, and the performances of the application itself, confirming the potential toward fulfilling objectives in a new area for the OPC UA protocol.

The real-time capabilities of OPC UA publish–subscribe-based applications are analyzed from different perspectives in studies such as [7,8], with various results and achievements and with respect to the TSN technology. As stated in [31], the concept of OPC UA publish–subscribe is to utilize the TSN extension of the Ethernet to obtain real-time guarantees for all the specific pub/sub processes and achieve a deterministic behavior regarding the transport of DataSetMessages. For the achievement of a complete real-time behavior between different devices, for avoiding desynchronization events, and for fulfilling the full potential of the OPC UA pub/sub mechanism, TSN technology represents the complete solution even if additional synchronization methods have been developed for certain scenarios [8]. For the present case, the obstacles that may occur at lengthy transmissions and that can alter the final output of the application, are relevant for understanding the need for TSN, and improvements in terms of performance must be expected with future integration between OPC UA and TSN.

In Table 1, the comparison of the relevant outcomes for all the case studies can be observed, with advantages and disadvantages for each version of the current study in the designated context.

With the successful transmission of images over the OPC UA publish–subscribe mechanism, the applicability of the OPC UA protocol extends to new functionalities and confirms the high applicability of the publish–subscribe paradigm in future developments with the possibility of achieving industrial demands. The current performances and the real-time behavior can be improved in the context of TSN technology. This provides ways of implementing applications with high capabilities in exchanging information at high rates between different devices over Ethernet, assuring the necessary objectives toward IIoT and Industry 4.0.

## 5. Discussion and Conclusions

The main conclusions of the case studies are that the OPC UA protocol can provide solutions toward image transmission in real-time scenarios and can achieve feasible performances using robust and scalable concepts. The publish–subscribe mechanism is reliable for multiple complex scenarios and with the current implementation, the long-term data transmission is analyzed under different circumstances. The applicability of the present strategies and architecture regarding multi-channel transmissions allows a faster image transmission, making possible the extension toward fields such as automotive industrial manufacturing and car-to-infrastructure communications, which are areas where OPC UA can still expand and provide significant improvements.

The objectives of the current work have been accomplished. In terms of real-time constraints over high data-volumes, the OPC UA pub/sub mechanism proved to be reliable, desynchronization risks being present at fast publishing intervals over long periods of time in the absence of TSN technology. The multi-channel UDP communication strategy allowed the achievement of transmission time intervals feasible in industrial scenarios, extending the applicability of the protocol. The implemented solution offers the possibility of image transmission over OPC UA in case studies with meaningful impact toward real use-cases with high demands, offering new approaches to the implementation of the pub/sub mechanism in a new context.

The main challenges for the present study are related to the multitude of vast scenarios that were needed for obtaining relevant details and observations regarding the factors that can increase or decrease the performance of the application. For each scenario and for each version of the application additional test code was implemented at all four steps of the transmission alongside particular functions that monitored the time periods of each transmission. The identification of the decisive criteria for the analysis of each case study proved to be a complex task due to the innovative aspect of the concept and the comparison with real case situations from the industry adds complexity to the implementation principles and expectations. The multi-channel over UDP communication strategy represents a scaled one-to-one scenario between the publisher and the subscriber that share the same channel. The main challenging issue for this type of strategy is the resource usage for all the developed channels, however, the implementation produces significant progress regarding the time of the transmission and the stability of the application.

Even with successful results of the OPC UA pub/sub image transmission application, the analysis of future development directions can lead to increased performances and stability. The most important steps that can be made in future studies are the implementation of different strategies regarding the image format and pixel manipulation for obtaining different ways of composing the payload and strategies regarding the structure and transmission sequences of the DataSetMessages. The adoption of the TSN technology can produce a more accurate view of the deterministic behavior, and in terms of hardware, the study concerning the capabilities of different industrial devices for OPC UA pub/sub based applications could increase the feasibility of the concept in industrial frameworks.

The approach of the OPC UA publish–subscribe mechanism in the context of image transmission expands the possibilities of usage for the well-defined OPC UA architectures and mechanisms and confirms the capabilities of multi-channel transmissions, taking into consideration delivery time intervals specific to industrial scenarios from different areas and providing the next step toward interoperability in the Industrial Internet of Things topic.

## Figures and Tables

**Table 1:** Outcome analysis for the case studies.

Case Study	Number of Pub-Sub Channels	Total Time for Full Transmission of an Identical Image	Factors That Can Produce Instability	Conclusions
1	1	64.5 min for phase 1 (publishing interval of 5 ms/pixel value) 3.12 min for phase 2 (publishing interval of 4 ms/pixel value)	- high volume of information needed to be transmitted by 1 channel - high length of the transmission increase the probability of desynchronization between devices	not feasible for industrial processes
2	4	12 s (publishing interval of 1 ms/pixel value)	- lower volume of information needed to be transmitted by 1 channel - not guaranteeing a low probability of desynchronization between devices	improved performances but far from the desired outcome
3	20	2.4 s (publishing interval of 1 ms/pixel value)	- adequate volume of information needed to be transmitted by 1 channel - guaranteeing a very low probability for desynchronization between devices	feasible in industrial scenarios for specific processes

## Data Availability

Not applicable.
